# Hydrothermal Processing and In Vitro Simulated Human Digestion Affects the Bioaccessibility and Bioactivity of Phenolic Compounds in African Pumpkin (*Momordica balsamina*) Leaves

**DOI:** 10.3390/molecules26175201

**Published:** 2021-08-27

**Authors:** Siphosanele Mafa Moyo, June C. Serem, Megan J. Bester, Vuyo Mavumengwana, Eugenie Kayitesi

**Affiliations:** 1 Department of Biotechnology and Food Technology, Faculty of Science, Doornfontein Campus, University of Johannesburg, P.O. Box 17011, Doornfontein, Johannesburg 2028, South Africa; 2 Department of Consumer and Food Sciences, University of Pretoria, Private Bag X20, Hatfield, Pretoria 0028, South Africa; 3 Department of Anatomy, Faculty of Health Sciences, University of Pretoria, Private Bag X323, Arcadia, Pretoria 0007, South Africa; june.serem@up.ac.za (J.C.S.); megan.bester@up.ac.za (M.J.B.); 4 Department of Biomedical Sciences, Division of Molecular Biology and Human Genetics, Faculty of Medicine and Health Sciences, Stellenbosch University, P.O. Box 19063, Tygerberg, Cape Town 7505, South Africa; vuyom@sun.ac.za

**Keywords:** leafy green vegetables, African pumpkin, phenolic compounds, bioaccessibility, antioxidant activity, in vitro digestion

## Abstract

The African pumpkin (*Momordica balsamina*) contains bioactive phenolic compounds that may assist in reducing oxidative stress in the human body. The leaves are mainly consumed after boiling in water for a specific time; this hydrothermal process and conditions of the gastrointestinal tract may affect the presence and bioactivity of phenolics either positively or negatively. In this study, the effects of hydrothermal processing (boiling) and in vitro simulated human digestion on the phenolic composition, bioaccessibility and bioactivity in African pumpkin were investigated in comparison with those of spinach (*Spinacia oleracea*). A high-resolution ultra-performance liquid chromatography, coupled with diode array detection, quadrupole time-of-flight and mass spectrometer (UPLC-DAD-QTOF-MS) was used to profile phenolic metabolites. Metabolites such as 3-caffeoylquinic acid, 5-caffeoylquinic acid, 3,4-dicaffeoylquinic acid, 3,5-dicaffeoylquinic acid and 4,5-dicaffeoylquinic acid were highly concentrated in the boiled vegetable extracts compared to the raw undigested and all digested samples. The majority of African pumpkin and spinach extracts (non-digested and digested) protected Deoxyribonucleic acid (DNA), (mouse fibroblast) L929 and human epithelial colorectal adenocarcinoma (Caco-2) cells from 2,2′-Azobis(2-methylpropionamidine) dihydrochloride (AAPH)-induced oxidative damage. From these results, the consumption of boiled African pumpkin leaves, as well as spinach, could be encouraged, as bioactive metabolites present may reduce oxidative stress in the body.

## 1. Introduction

*Momordica balsamina*, also known as African pumpkin or balsam apple, is a wild leafy vegetable of the family Cucurbitaceae [1]. The species is mainly consumed in Africa and Asia and has little commercial value; however, it is a notable nutritional and medicinal plant that has been found to possess antidiabetic and antiulcerogenic properties in tests on diabetic rats and rats with induced gastric ulceration [2,3]. This plant may also have anti-cancer properties where 10–20 µg/mL reduced the viability of breast (MCF-7) cancer cells [4]. 

Fruit and vegetable consumption is associated with the reduced development of cardiovascular disease and protection against diet-related chronic diseases [5]. The health benefits presented by fruits and vegetables have been widely identified to be due to the presence of antioxidants and bioactive compounds such as phenolic compounds and carotenoids [6]. Compounds such as 4-caffeoylquinic acid and rutin have previously been identified in the African pumpkin [7,8], while phenolic contents of 0.49 mg GAE/g of dry extract and 39.8 mg GAE/g of the dry extract have been reported [9,10]. These compounds may exert their health benefits in the gastrointestinal tract or may be absorbed into the circulatory system [5]. It is known that phenolic stability and associated activity may be affected by different processing methods. Studies have shown that thermal treatments used to cook vegetables prior to consumption may cause chemical and structural changes to polyphenols and may have positive or negative effects on them [11].

When consumed, phenolic compounds in vegetable material are released and solubilized into the gastrointestinal digesta (bioaccessibility) with in situ or ex situ effects, and/or can be absorbed into the bloodstream [5]. The concentration of phenolics bioaccessible in the intestinal tract may differ from the concentration in the native food matrix [12]. Therefore, it is important to investigate the effects of digestion on the bioaccessibility and bioactivity of phenolic compounds. In vivo methods, using humans or animals, are suitable but are also highly variable, complex, laborious, expensive, and come with several ethical restrictions [13]. The in vitro method (with limited ethical restrictions) can be better used to simulate human digestion and is considered technically simple, fast, safe, and relatively inexpensive [14]. Simulated digestion can be utilised to investigate isolated compounds or homogenised foods in a closed system, and subsequent analysis of the amount of soluble compound and bioactivity existing in the supernatant obtained by filtration or centrifugation [15] can be evaluated.

No studies have investigated the effect of boiling and in vitro digestion on the bioaccessibility of phenolic compounds and bioactivity from raw and boiled leaves of the African pumpkin. Spinach was used as a control as it is widely consumed, whereas the African pumpkin is underutilised in comparison. The results of this study might be useful in promoting more studies into the African pumpkin in human diets and may provide a reference for further use in value-added products with African pumpkin leaves.

## 2. Results and Discussion

### 2.1. Effect of Boiling and In Vitro Digestion on Compounds Identified in Raw and Boiled AFRICAN Pumpkin by UPLC-QTOF-MS

Table 1 shows a total of 15 metabolites identified from undigested and in vitro digested extracts of African pumpkin. Mostly, chlorogenic acids (3-caffeoylquinic acid, 4-caffeoylquinic acid, 5-caffeoylquinic acid, 3,4-dicaffeoylquinic acid, 3,5-dicaffeoylquinic acid, 4,5-dicaffeoylquinic acid) and flavonoids (catechin, myricitin, quercetin-3-*O*-robinobioside, rutin, quercetin-3-*O*-glucoside) were identified. Compounds such as 4-caffeoylquinic acid and rutin have been previously identified in African pumpkin [7,8]. Similar species of African pumpkin such as *Momordica dioica*, *Momordica charantia*, and *Momordica charantia* var. *muricata* have been found to contain gallic acid, chlorogenic acid, rutin, caffeic acid, ferulic acid, quercetin, ellagic acid and catechin [16]. The presence of all identified metabolites was significantly affected by boiling and in vitro digestion, and therefore, the principal component analysis (PCA) model was used to depict the presence of metabolites affected by boiling and digestion.

**Figure 1:**
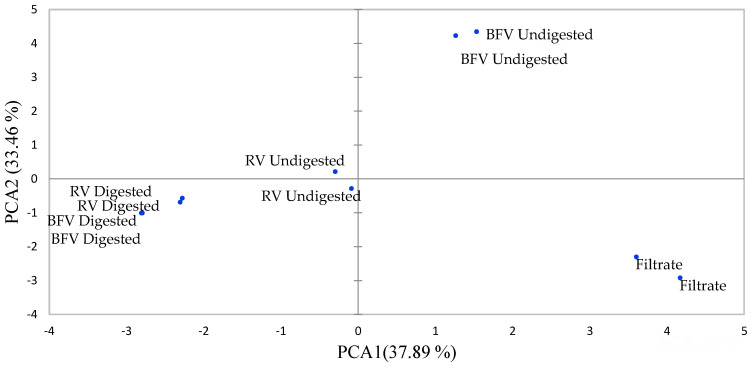
Principal component analysis (PCA) scores of the African pumpkin, RV-Raw Vegetable, BFV-Boiled Filtered Vegetable.

**Figure 2:**
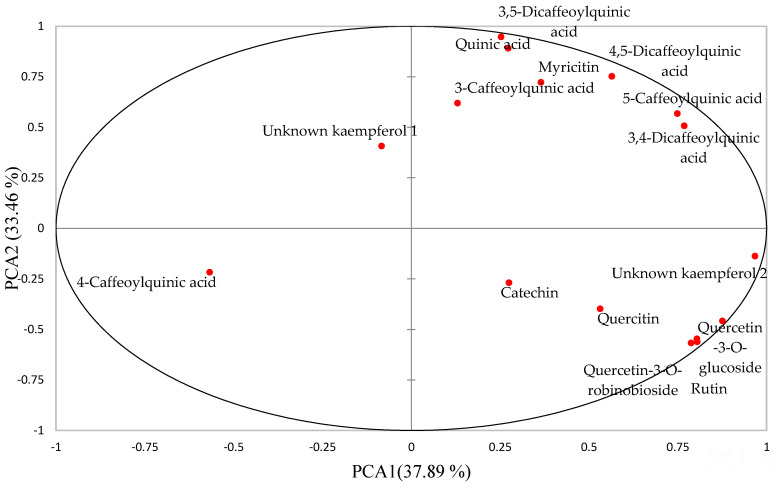
Principal component analysis (PCA) loadings of African pumpkin phenolic compound concentration.

As shown in Figure 1 and Figure 2, the first two principal components (PCs) explained 71.34% of the total variation. PC-1 accounted for 37.89% of the variation while PC-2 explained 33.46% of the variation. The PC-1 positive axis differentiates the filtrate sample and undigested extracts of boiled filtered vegetable (BFV) from the samples on the negative axis, that is, the digested raw vegetable (RV), digested BFV and undigested RV extracts. The positive axis of PC-1 indicates a higher concentration of phenolic metabolites than the negative axis, indicating that the filtrate sample and undigested extracts of BFV had a higher concentration of phenolics than RV, BFV digested and RV undigested extracts. The process of boiling African pumpkin leaves increased the extractability of phenolics from cellular components through the destruction of the cellular matrix. Further, the leaching of phenolics into the boiling water occurred, revealing the susceptibility of quercetin-3-*O*-robinoside, rutin, quercetin, quercetin-3-*O*-glucoside, catechin, and unknown kaempferol 2 to leaching, as they are polar compounds. These results, therefore, prompted the addition of a sample with the filtrate retained, (BV-NF), to the rest of the analysis. The association of 3-caffeoylquinic acid, 5-caffeoylquinic acid, 3,4-dicaffeoylquinic acid, 3,5-dicaffeoylquinic acid, 4,5-dicaffeoylquinic acid with BFV undigested extracts revealed their thermostability. 5-caffeoylquinic and 3,5-caffeoylquinic acids are quite stable when heated to 100 °C in water [17].

The PC-2 negative axis separated digested extracts from undigested extracts of BFV in the positive axis. Fewer of the phenolics were concentrated in the negative axis, indicating the degradation of phenolics during digestion. The 4-caffeoylquinic acid remained highly concentrated in the digested extracts. In another study, 3-caffeoylquinic acid remained the most abundant phenolic after digestion of mulberry (*Morus alba* L.) leaves [18]. Phenolic compounds are highly sensitive to gastrointestinal pH changes and enzyme interaction and can be oxidised, hydrolysed, deglycosylated, and transformed into metabolites different from their parental compounds [19]. The leaves of the Madeiran elderberry (*Sambucus lanceolata*) depicted decreased concentrations of 3-*O*-caffeoylquinic acid, 5-*O*-caffeoylquinic acid, and rutin after in vitro digestion with losses of 53%, 70%, and 70% being recorded, respectively [20].

### 2.2. Effect of Boiling on TPC and TFC of African Pumpkin and Spinach

Table 2 illustrates the total phenolic content (TPC) and total flavonoid content (TFC) of African pumpkin and spinach, expressed in mg GAE/g dw and mg QE/g dw, respectively. The RV (raw vegetable) undigested extracts exhibited a TPC and TFC of 64.53 ± 9.1 and 69.00 ± 1.3, respectively. Results from other studies are not comparable with the current study, (with values of 0.49 and 39.8 TPC expressed as mg GAE/g of dry extract [9,10]). Differences in phenolic contents could be ascribed to the different extraction techniques and environmental conditions in which the plants were sampled.

Heat treatments can contribute to the softening of vegetable tissues and subsequent extraction of phenolic compounds from the cellular matrix [21], and therefore, losses or gains in phenolic contents could be experienced. In this study, as shown in Table 2, boiling did not significantly alter the phenolic contents of the African pumpkin leaves, as similar TPC (mg GAE/g dw), values of 64.53 ± 9.1, 60.41 ± 15 and 52.27 ± 2.0 were obtained for RV, BFV (boiled filtered vegetable), and BV-NF (boiled not filtered vegetable), respectively. On the contrary, TFC of the African pumpkin was significantly (*p* < 0.05) reduced after boiling due to filtering of the boiled water, as a TFC of 44.34 ± 1.2 mg GAE/g dw was observed for BFV as compared to 63.90 ± 5.6 mg GAE/g dw, and 69.00 ± 1.3 mg GAE/g dw for BV-NF and RV, respectively. The extracted flavonoids, therefore, leached into the boiling water because they are polar compounds. For spinach, both the TPC and TFC were significantly (*p* < 0.05) reduced after boiling, with no significant differences noted for BFV and BV-NF. This suggests the thermal liability of spinach phenolics, rather than losses occurring due to leaching. Decreases in TPC from 71.67 mg/g to 61.68 mg/g after thermal treatment have also been observed by Amin et al. [22]. The manner in which plant phenolics are affected may depend on the chemistry of the plant matrix [23]. Vegetables such as kale (*Brassica oleracea*) showed decreases in TPC from 94.78 mg GAE/100 g fresh weight (fw) to 79.82 mg GAE/100 g fw after boiling [24], while *Brassica Juncea* revealed increases from 23.10 mg GAE/100 g fw to 27.70 mg GAE/100 g fw [25]. The surface morphology of the vegetable may be altered after boiling, indicating the destruction of cell walls and release of phenolic compounds from cell wall components.

### 2.3. Effect of In Vitro Digestion on TPC and TFC of African Pumpkin and Spinach

Plant foods are subjected to the conditions of the gastrointestinal tract after consumption. Therefore, phenolic compounds present may be highly metabolised through oxidation, hydrolysis, and transformation into compounds different from the initial metabolites [26]. Prior to exerting antioxidant activity in the digestive tract and absorption in the intestine, phenolic metabolites should be bioaccessible (released from plant matrices and subsequent solubilization in the gastric and intestinal phase digesta [5]).

As depicted in Table 2, the TPC of RV African pumpkin extracts was not affected by digestion, with bioaccessibility remaining constant at 57%, 57%, and 69% for oral, gastric and duodenal digestion, respectively. However, the flavonoid content of RV African pumpkin extracts significantly (*p* < 0.05) improved, translating to an improved bioaccessibility from 6%, 43%, and 72% for oral, gastric and duodenal, respectively. For the BFV samples, the TPC did not change throughout digestion (oral, gastric, and duodenal) with a constant release of 71%, 67%, and 73% of phenolics, respectively. In contrast, the TFC of the African pumpkin BFV extracts increased significantly (*p* < 0.05) from oral, gastric to duodenal digestion at 42%, 64%, and 88%, respectively.

In accordance with our results, Gonçalves et al. [27] observed no change in TPC during digestion of the common plantain (*Plantain major*) and golden thistle (*Scolymus hispanious*). For the BV-NF extracts of African pumpkin, both the TPC and TFC were significantly (*p* < 0.05) reduced after digestion, with a final bioaccessibility of 7% and 5% at the duodenal stage, respectively. Variance in bioaccessibility results with respect to the samples concerned (RV, BFV, and BV-NF) could be attributed to the differences in the presence of simple and highly polymerised metabolites [27,28]. Factors such as chemical structure modification, reduced or increased stability and association with other molecules (fibres, protein, and sugars) within the digesta may influence the bioaccessibility of phenolics [29].

For the RV extracts of spinach, both the TPC and TFC significantly (*p* < 0.05) reduced, with bioaccessibility reducing from 71% and 51% for oral digestion to 35% and 21% for duodenal digestion, respectively. The BV-NF extracts of spinach also had a significant (*p* < 0.05) reduction in TPC and TFC after digestion, with duodenal extracts recording a bioaccessibility of 23% and 18%, respectively. No significant reductions in TPC and TFC of spinach BFV extracts were observed after digestion, although significant (*p* < 0.05) spikes in TPC occurred during gastric digestion from 41% to 72%. Gastric acidic conditions have been reported to influence the increased stability of phenolic compounds and improved release from the food matrix [30]. Phenolics are commonly present in plant tissues as insoluble forms and soluble conjugates which are covalently bound to cell wall structural components and sugar moieties, and therefore, acid treatment may break glycosidic bonds and solubilize sugars while not affecting ester linkages [31]. Decreases are experienced after gastric digestion, as phenolics are sensitive to the alkaline conditions of the duodenal phase; complexes could be formed between extracted phenolics and metal ions, fibre, and proteins [32].

### 2.4. Effect of Boiling on Radical Scavenging Activity/Redox Potential of African Pumpkin and Spinach

Antioxidants play a pivotal role in the prevention of disease and maintenance of health [33]. Plant foods act as dietary sources of antioxidants, which include phenolic compounds, minerals (Se, Zn), and vitamins (β-carotene, vitamin C, and E) [34]. Antioxidants may function as hydrogen donors, reducing agents, quenchers of singlet oxygen, and metal chelators. Therefore, Trolox equivalence antioxidant capacity (TEAC), ferric reducing antioxidant power (FRAP) and oxygen radical antioxidant capacity (ORAC) assays were utilised in this study to assess the radical scavenging activity, reduction potential and hydrogen donation properties of the African pumpkin and spinach, respectively.

As shown in Table 3, the boiling method utilised in this study did not significantly change the TEAC radical scavenging properties of the African pumpkin and spinach undigested extracts. In addition, the process of discarding the filtrate did not significantly cause a loss in TEAC activity of the African pumpkin and spinach undigested extracts. This suggests that compounds responsible for the radical scavenging activity of both vegetables are highly stable under the boiling conditions employed in this study, with low solubility into the cooking water. In other studies, decreases in TEAC activity from approximately 140 µM TE/g dw to 105 µM TE/g dw have been reported for spinach [35], while no changes in TEAC activity were reported after boiling of kale, with the activity of 119.01 ± 0.41 µM TE/g dw and 119.72 ± 1.19 µM TE/g dw for raw and boiled vegetable, respectively [36]. Positive correlations (Supplementary Table S1) were observed between TPC, TFC and TEAC activity for both African pumpkin and spinach, suggesting phenolic metabolite involvement in radical scavenging activities recorded in this study.

For FRAP activity, the reducing effect of raw undigested African pumpkin (50.14 ± 6.0 µM TE/g dw) was improved by boiling the vegetable, accompanied by retaining of the filtrate as represented by the BV-NF undigested extract at 148.78 ± 16 µM TE/g dw. Removal of filtrate after boiling as represented by the BFV undigested extract (48.36 ± 11 µM TE/g dw) caused a reduction in FRAP activity to a similar initial value to that of the raw extract. For the spinach extracts, FRAP activity did not significantly change after boiling and retaining filtrate with values showing a small increase from 334.16 ± 4.5 µM TE/g dw for RV undigested extract to 351.83 ± 2.9 µM TE/g dw for undigested BV-NF extract. However, a large reduction of FRAP activity was observed after boiling and removal of the filtrate (undigested BFV extract 263.46 ± 0.96 µM TE/g dw) from spinach. Therefore, the leaching of antioxidant metabolites into cooking water may contribute to a reduction in the reducing capacity of the African pumpkin and spinach if the filtrate is not retained. Losses in antioxidant activity from 1277.48 ± 44.41 µmol Fe(II)/100 g for fresh kale to 326.58 ± 25.17 µmol Fe(II)/100 g for boiled kale have been reported [37]. Losses were attributed to a combination of effects, such as leaching of water-soluble antioxidants, degradation of heat-sensitive antioxidant compounds and oxidative reactions [37]. In contrast, increases in FRAP activity were reported for Kaffir lime leaves (*Citrus hystrx*), with raw leaves possessing an activity of 447 ± 9.01 µmol FeSO_4_/g and boiled leaves 583 ± 18.17 µmol FeSO_4_/g [38].

Boiling did not significantly alter the hydrogen donation property of the African pumpkin undigested extract, as determined by the ORAC method. For spinach, the hydrogen donation property was improved by boiling, with no significant differences noted between BFV and BV-NF undigested extracts. Therefore, retaining the filtrate does not necessarily improve the hydrogen donating properties of spinach nor that of the African pumpkin. Increases in ORAC activity after boiling has been reported for *Amaranthus hybridus* from 1231.3 ± 48 µmol TE/kg fw to 3161.01 ± 93 µmol TE/kg fw [39]. Negative correlations were observed between TPC, TFC and ORAC activity at (r) of −0.657 and −0.771 for the African pumpkin and −0.771 and −0.600 (Supplementary Table S1) for spinach, respectively. Therefore, other metabolites that might be present in the extracts such as β-carotene and vitamin C might have contributed to the hydrogen donating capabilities of the African pumpkin and spinach extracts.

### 2.5. Effect of In Vitro Digestion on Radical Scavenging Activity/Redox Potential African Pumpkin and Spinach

In addition to the effects of boiling on the antioxidant activity of the African pumpkin and spinach, digestion conditions involving changes in pH and enzyme activity may have influenced the resultant bioactivity of the digests. As depicted in Table 3, the TEAC radical scavenging activity of raw African pumpkin extracts did not significantly decrease after complete digestion, with a loss of about 17% compared to the undigested extract. For raw spinach, significant (*p* < 0.05) decreases (loss of 51%) in TEAC activity were recorded after complete digestion. Yang et al. [40], reported 40% losses in TEAC activity after gastrointestinal digestion of the herb Basil. For the BFV extracts, losses of about 8% and 48% for the African pumpkin and spinach, respectively, were obtained after complete digestion, although losses of 8% were not significant for the African pumpkin digests. Boiled samples with the filtrate retained, i.e., the BV-NF extracts, experienced the highest losses in TEAC activity after complete digestion for both the African pumpkin and spinach at 117% and 84%, respectively. This suggests that the presence of metabolites in the filtrate renders them highly sensitive to pH changes and easily accessible to interaction with digestive enzymes or other food components, therefore changing their molecular weight, chemical structure, and solubility [32].

For FRAP activity, the African pumpkin raw extracts depicted insignificant losses of about 5% after complete digestion, while spinach raw extracts exhibited significant (*p* < 0.05) losses of 32% after complete digestion. In another study, leafy vegetable link (*Leucas aspera*) exhibited 50% loss in FRAP activity after intestinal digestion compared to the undigested extract [41]. For the spinach BFV extracts, significant gains (*p* < 0.05) of about 37% occurred after complete digestion, whereas with the African pumpkin BFV extracts, gains of about 142% in FRAP activity were observed after complete digestion. The improved FRAP activity could be attributed to the deprotonization of the hydroxyl groups on the aromatic rings of the phenolics [6]. At higher pH (characteristic of the duodenal phase), the deprotonation of the phenolic compound groups with terminal -OH offers strong binding sites to form a stable complex with Fe^3+^ [42]. For the BV-NF extracts, significant (*p* < 0.05) losses of 125% and 65% occurred after complete digestion. Compounds in BV-NF were probably easily oxidised, hydrolysed, or transformed to other compounds with less antioxidant activity. In addition, they could have conjugated with other molecules such as metal ions, proteins, and fibre.

For the ORAC hydroxyl donating activity, the raw African pumpkin showed significant (*p* < 0.05) losses of 47% after complete digestion, while the spinach samples revealed significant (*p* < 0.05) gains of 32%. Different ORAC activities between the African pumpkin and spinach could probably be due to the chemodiversity of the phenolic compounds of these vegetables, ranging from simple to highly polymerised molecules [28] which could be affected differently during digestion. Yang et al. [40] reported no significant changes in ORAC activity for Basil after complete digestion. For the BFV extracts of both the African pumpkin and spinach, no significant losses were observed at 20% and 17%, respectively. Significant (*p* < 0.05) losses of 44% and 52% were observed for the African pumpkin and spinach extracts of the BV-NF sample after complete digestion. Significant (*p* < 0.05) positive correlations were observed between TFC and TEAC, FRAP for the African pumpkin (Supplementary Table S1) while for all other extracts from the African pumpkin and spinach moderate to poor correlations were observed. Phenolic compounds together with other compounds not investigated in this study such as vitamins and minerals also could have contributed to the antioxidant activity measured.

### 2.6. Cellular Antioxidant Activity

As shown in Table 4, all undigested extracts of African pumpkin and spinach completely protected L929 and Caco-2 cells from AAPH induced oxidative damage; in addition, boiling of the vegetable did not significantly affect the protective abilities of either the African pumpkin or spinach.

The protective ability of the African pumpkin was reduced for all raw digested (oral, gastric, and duodenal) extracts at 78%, 55%, and 64% oxidative damage, respectively, for the L929 cells. For the physiologically relevant Caco-2 cells, all raw African pumpkin digested extracts (but not oral digests) completely prevented 2,2′-Azobis(2-methylpropionamidine) dihydrochloride (AAPH) induced oxidative damage. For spinach, all raw digests provided complete protection against oxidative damage to both L929 and Caco-2 cells, with the exception of the oral digests, where slight oxidative damage was observed at 16% for L929 cells and a higher oxidative damage than the control (AAPH + Caco-2 cells + Phosphate-buffered solution (PBS)) at 102.05%. All boiled digested extracts from spinach, i.e., BFV and BV-NF, completely protected L929 and Caco-2 cells from oxidative damage, with the exception of BFV and BV-NF oral digests as well as gastric digests from BV-NF, which exhibited slight oxidative damage at 10%, 6.6%, and 10.7%, respectively. All BV-NF digested extracts from the African pumpkin completely protected L929 and Caco-2 cells from oxidative damage. Slight oxidative damage occurred for L929, and Caco-2 cells incubated with African pumpkin BFV, with the exception of gastric digests, which showed complete protection for L929 cells, and duodenal digestion, which depicted a higher oxidative damage than the control (AAPH + L929 cells + PBS) at 114%. In light of these data, it was observed that retaining the filtrate after boiling of African.

Pumpkin improves its protective ability against oxidative damage throughout the digestion phases. Indeed, the majority of extracts prevented oxidative damage in the duodenal phase, particularly with the Caco-2 cells. This is significant, as the gastrointestinal tract is constantly exposed to reactive species [43]. In other studies, Broccoli sprouts at 1 mg/mL reduced the development of reactive oxygen species (ROS) by 42.92% and 76.59% for non-digested and gastrointestinal digested extracts, respectively, in a non-transformed, non-tumorigenic colon (NCM460) cell line [44]. The antioxidant properties of foods rarely exist due to only one source of compounds, but rather from the synergistic effect of different compounds/molecules such as proteins, vitamins, Maillard reaction products, phenolic compounds, as well as digestion products [45].

Negative correlations indicate the role of phenolics in preventing oxidative damage, and in addition, negative correlation between radical scavenging activity assays and 2′,7′-dichlorodihydrofluorescein diacetate (DCFH-DA) assays implies the involvement of electron-donating mechanism and hydrogen transfer mechanism in preventing AAPH induced oxidative damage. Negative strong correlations were only obtained for spinach between ORAC assay and DCFH-DA_L929_ after gastric digestion (Supplementary Table S1). Therefore, hydrogen transfer mechanisms could have been involved in preventing oxidative damage.

### 2.7. Macromolecule Protective Ability

#### 2.7.1. Copper-Mediated Human LDL Oxidation

**Figure 3:**
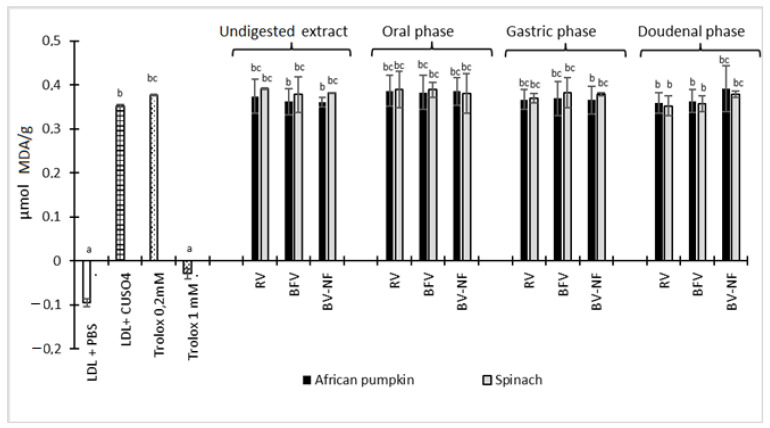
Inhibition of copper-mediated human LDL oxidation by African pumpkin and spinach extracts. LDL + PBS contains LDL and phosphate-buffered solution, LDL + Cu^2^+ contains LDL and Cu^2^+ only. All samples contain LDL and Cu^2^+. Raw vegetable (RV), boiled filtered vegetable (BFV), boiled vegetable—not filtered (BV-NF). Trolox 0.2 mM and 0.4 mM were used as reference antioxidant standards. Alphabets in superscripts on bars show significant differences at *p* < 0.05.

Low-density lipoproteins (LDLs) are the major carriers of cholesterol in body tissue, and when oxidised it is involved in the development of atherosclerosis by promoting inflammation and lipid deposition in the arterial wall [46]. As depicted in Figure 3, none of the extracts from African pumpkin and spinach was able to inhibit the oxidation of LDL induced by Cu^2+^.

In previous studies [47,48], *Bidens pilosa* and *Solanum nigrum* protected LDL from Cu^2+^ induced LDL damage, but *Momordica balsamina* did not possess the same characteristics. This suggests that some vegetables possess the ability to protect LDL oxidation, probably due to the diversity and concentration of bioactive metabolites present. In another study, sweet potato (*Ipomoea batatas* L.) leaves were found to protect LDL from 95% 2,2′-azobis-methoxy-2,4-dimethylvaleronitrile (AMVN) induced damage due to the content of caffeoylquinic acid derivatives [49]. Although the African pumpkin also contains caffeoylquinic acid derivatives (shown in Table 1), it did not protect LDL from oxidative damage. However, the type and concentration of compounds together with the synergistic effects of these caffeoylquinic derivatives with other compounds could play a role in LDL protection.

#### 2.7.2. Inhibition of AAPH-Induced Oxidative DNA Damage

Deoxyribonucleic acid (DNA) is one of the most important targets for ROS in regard to the development of cancer and at the molecular level, and its damage may come in many forms [50]. In this study, the damage of PBR322 DNA was observed by its conversion from a native supercoiled form to a more linear form through single or double-strand breaks.

**Figure 4:**
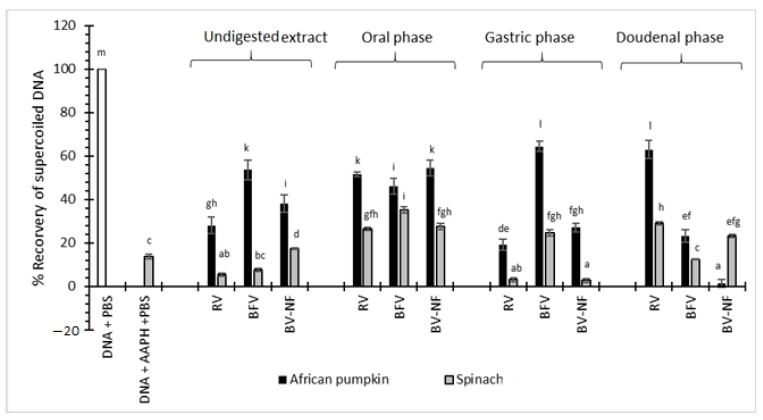
% Recovery of supercoiled DNA. DNA + PBS—DNA mixed with 0.1 M phosphate-buffered solution (PBS), DNA + AAPH—DNA mixed with 1 mg/mL AAPH. Raw vegetable (RV), boiled filtered vegetable (BFV), boiled vegetable—not filtered (BV-NF). Alphabets in superscripts on bars show significant differences at *p* < 0.05.

Figure 4 shows the % recovery of supercoiled DNA after incubation of vegetable samples with AAPH and DNA. With the undigested extracts, the boiling of African pumpkin and spinach significantly (*p* < 0.05) improved the ability of the vegetable to protect DNA from AAPH-induced damage. For spinach, retaining the filtrate after boiling was beneficial, as significant differences (*p* < 0.05) existed between BFV and BV-NF samples. The filtrate of spinach might contain beneficial compounds leached from the vegetable material during boiling that are essential for DNA protection. In contrast, the filtering of African pumpkin after boiling significantly (*p* < 0.05) improved its protective abilities. The filtrate from African pumpkin might contain toxic compounds such as oxalates, which have been reported to contribute to DNA damage [51]. In another study, vegetable powders of Chinese plantain (*Plantago asiatica*), and Chameleon plant (*Houttuyni acordata*) protected DNA from H_2_O_2_ induced damage in human lymphocytes [52].

For the raw African pumpkin digested extracts, DNA protective ability was significantly (*p* < 0.05) improved after oral and duodenal digestion from 28.08% to 51.67% and 62.96%, respectively, compared to the undigested extracts. Similarly, the spinach raw digested extracts protected DNA from oxidative damage at 26.4% and 29.04% for oral and duodenal digests, respectively. The presence of low pH in the gastric phase might have contributed to the significant losses in protective ability towards DNA, with the recovery of supercoiled DNA of 19.05% and 3.07% for raw African pumpkin and raw spinach, respectively.

For the African pumpkin BFV and BV-NF extracts, the protective ability against DNA damage was significantly (*p* < 0.05) reduced for duodenal digests, from 53.58% to 23.19% for BFV and 38.06% to 1.28% for BV-NF extracts compared to the undigested extracts. For spinach, the opposite was observed, as boiled extracts (BFV and BV-NF showed improved recovery of supercoiled DNA from 7.6% to 12.31% for BFV and 17.39% to 23.30% for BV-NF compared to the undigested extracts. These results, therefore, suggest that due to the chemo-diversity of metabolites in African pumpkin and spinach, their ability to prevent DNA damage is affected differently after boiling and in vitro digestion. The stability of compounds responsible for DNA protection varies according to plant species and plant matrix. The protective ability of RV, BFV, and BV-NF of spinach were improved after digestion, whereas for the African pumpkin boiling reduced the protective ability of BFV and BV-NF after digestion. Strong positive correlations were only obtained after the duodenal digestion of African pumpkin for TFC at 0.886. All other extracts, as depicted in Supplementary Table S1, showed moderate to poor correlations.

## 3. Materials and Methods

### 3.1. Chemicals and Reagents

HPLC grade reagents were used in this study. The following reagents were supplied by Sigma-Aldrich Pty. Ltd. (Johannesburg, South Africa): Folin-Ciocalteu reagent, Trolox, quercetin, gallic acid α-amylase from *Aspergillus oryzae* (30 U/mg protein), pepsin from porcine gastric mucosa (250 units/mg solid), pancreatin from porcine pancreas (8 × USP specification), and low-density lipoprotein (7.6 mg protein/mL) from human plasma. PBR322 vector DNA (0.5 µg/µL) was supplied by Promega (Madison, WI, USA).

### 3.2. Sample Preparation

Selected vegetable species were sampled from the Limpopo region of South Africa in the village of Tzaneen, and confirmed by Professor A. Moteetee from the Department of Botany, University of Johannesburg. Harvested vegetables were washed and boiled in 1 L dH_2_O for 500 g vegetable for 20 min and further freeze-dried for 24 h at −55 °C. Samples prepared as shown in Supplementary Figure S1 were raw vegetable (RV), boiled filtered vegetable (BFV), boiled vegetable–not filtered (BV-NF), and the filtrate (F).

### 3.3. Methanolic Extraction for Undigested Samples

Undigested extracts were prepared according to Moyo et al. [53] by combining 2 g of powdered leaf material with 20 mL of 80% methanol and 1% formic acid. The mixture was sonicated for 10 min, then centrifuged for 10 min at 1395× *g* and 4 °C. The resultant supernatant was evaporated to 1 mL using a Buchi rotary evaporator and freeze-dried. The extracts were stored at −20 °C. For reconstitution, Phosphate-buffered solution (PBS) was used for all experiments, while (methanol:DMSO) at a ratio of 50:50 was used to reconstitute extracts for LC-MS analysis.

### 3.4. Human Simulated Gastrointestinal Digestion

To simulate human digestion, a three-phase method was used according to Moyo et al. [47]. The oral digested extracts were prepared by homogenising 1 g vegetable fraction with 10 mL saline solution (pH 5.5), which consisted of KCl (5 mM), CaCl_2_ (6 mM) and NaCl (120 mM). 1000 units of α-amylase were included thereafter, and pH adjusted to 6.5 for incubation for 5 min in a 37 °C water bath, shaking at 95 rpm. The oral digests were then subjected to gastric digestion by the reduction of pH to 2.2 with 0.1 N HCl and the addition of 0.5 mL of a porcine pepsin solution at a concentration of 0.075 g/mL in 0.1 N HCl. Further, 15 mL of the saline solution was added, and the mixture was incubated at 37 °C for 2 h in a water bath, shaking at 95 rpm. The gastric digests were then subjected to duodenal digestion by mixing with 10 mL of 0.05 M phosphate buffer at pH 7.0 and 3.0 mL of duodenal juice, consisting of 2 g of pancreatin in 60 mL, 240 mL of 50 mM CaCl_2_, 12.5 g of bile salts, and 0.1 M of NaHCO_3_. The pH was adjusted to 7 by 1 M NaOH. Thereafter the volume was attuned to 40 mL with phosphate buffer and incubated for 2 h in a 37 °C water bath, shaking at 200 rpm. The duodenal digests were centrifuged for 15 min at 12,000× *g* and 4 °C, and the supernatant was used for all analyses.

### 3.5. Determination of Phenolic Composition

#### 3.5.1. Analytical Conditions for UPLC-DAD-QTOF-MS

Isolation and quantification of metabolites from the African pumpkin were achieved by the use of a high-resolution UPLC-MS, where the (UPLC) Waters Acquity ultra-performance liquid chromatography (Waters, Milford, MA, USA) was connected to a Waters Synapt G2 quadrupole time-of-flight (QTOF) mass spectrometer (MS). For the mobile phases, A consisted of 0.1% formic acid while B had 0.1% formic acid in acetonitrile, injected at a volume of 2 µL in a Waters HSS T3 column of 2.1 × 100 mm with 1.7 μm particle size (Waters, Milford, MA, USA). Initially, eluent A conditions were kept constant for 1 min at 100% chromatographic gradient. Subsequently, eluent B conditions were set for 22 min and 50 s at gradient 28% and 40%, respectively, with a 1.5 min wash step at 100% thereafter. The column was then brought back to the initial conditions for 4 min and the temperature was kept at 55 °C with a flow rate of 0.3 mL/min. Ideal experimental settings for the MS consisted of sample cone voltage of 15 V, desolvation temperature at 275 °C and desolvation gas at 650 L/h with negative mode electrospray ionization. Resolution mode and MSE mode was used to collect data at a mass range of *m*/*z* 150 to 1500. MSE mode had two channels consisting of a collision energy ramp of 40 to 100 V and low collision energy (4 V) to obtain retention times, the mass-to-charge ratio (*m**/**z*), fragmentation data, molecular formula, diagnostic fragments, and UV maxima. MS accuracy was ensured by calibrating the instrument with sodium formate and a lock mass standard (leucine enkephalin). Selected compounds were tentatively identified based on accurate mass, and comparison of the elemental compositions to databases such as Metlin and Chemspider. Reference calibrant rutin was used for compounds where no standards were available, to quantify compounds based on the areas of their extracted mass chromatograms.

#### 3.5.2. Determination of Total Phenolic Content

The TPC of vegetable extracts was analysed using the Folin-Ciocalteu (F-C) method according to Serem & Bester [54]. Briefly, 10 µL of a sample (1 mg/mL) followed by 50 µL F–C reagent (14X diluted) and 50 µL of 7.5% solution of sodium carbonate was added to a 96 well plate and absorbance read at 630 nm using a fluorescence plate reader (BMG lab technologies, Offenburg, Germany). Results were reported as mg gallic equivalent GAE/g dry weight (mg GAE/g dw).

#### 3.5.3. Determination of Total Flavonoid Content

The TFC of vegetable extracts was analysed using the aluminium chloride method according to Amaral et al. [55]. Briefly, a 10 µL sample (1 mg/mL), followed by 30 µL of 2.5% sodium nitrite, 30 µL of 1.25% aluminium chloride and 100 µL of 2% sodium hydroxide solution were added to a 96 well plate and absorbance read at 450 nm. Results were reported as mg quercetin equivalent QE/gram dry weight (mg QE/g dw).

#### 3.5.4. Estimation of Bioaccessibility

The percentage bioaccessibility (%BA) for TPC (%BA-TPC) and for TFC (%BA-TFC) after simulated gastrointestinal digestion was determined according to Seraglio et al. [28] using the following equation:

% BA-TPC = PCA/PCB × 100(1)

% BA-TFC = FCA/FCB × 100(2)

PCA is the TPC (mg GAE/g dw) in samples after simulated digestion and PCB is the TPC in undigested samples/before digestion (mg GAE/g dw).

FCA is the TFC (mg QE/g dw) in samples after simulated digestion and FCB is the TFC in undigested samples/before digestion (mg QE/g dw).

### 3.6. Determination of Radical Scavenging Activity/Redox Potential

#### 3.6.1. Trolox Equivalence Antioxidant Capacity (TEAC) or the ABTS Assay

The ABTS/TEAC radical scavenging assay was employed according to Serem & Bester [54]. The radicals of 2,2′-azino-bis(3-ethylbenzothiazoline-6-sulfonate) radical cation (ABTS•^+^) consisted of equal amounts of 8 mM ABTS and 3 mM potassium persulfate. The resultant solution was incubated in the dark for at least 12 h and used within 16 h. Briefly, a 10 µL sample (1 mg/mL) was combined with 290 µL ABTS working solution at 0.26 mM concentration and incubated for 15 min at 37 °C. Absorbance was read at 734 nm and results were reported as µmol Trolox Equivalent/g dry weight (TE/g dw).

#### 3.6.2. Ferric Reducing Antioxidant Power (FRAP) Assay

The ferric reducing antioxidant power assay followed the method of Moyo et al. [53]. The FRAP reagent was prepared by sequentially mixing 25 mL of acetate buffer, 2.5 mL of 2,4,6-Tripyridyl-S-triazine (TPTZ), and 2.5 mL of FeCl_3_·6H_2_O. The mixture was submerged and kept in a 37 °C water bath. Briefly, 90 µL volume of double-distilled water (ddH_2_O), 30 µL of a sample (1 mg/mL) was combined with 900 µL of the FRAP reagent and incubated for 30 min the dark. The resultant ferrous tripyridyltriazine-coloured complex was read at 595 nm using an absorbance reader (iMark microplate, Bio-Rad laboratories 168-1130). Results were expressed as µmol TE/g dw.

#### 3.6.3. Oxygen Radical Antioxidant Capacity (ORAC) Assay

Antioxidant activity was measured according to the modified method of Serem & Bester [54]. Briefly, 10 µL (1 mg/mL) sample, 165 µL of 0.139 mM fluorescein was added followed by 25 µL of 0.24 M AAPH. Samples were thoroughly mixed and incubated at 37 °C. Fluorescence was measured every min for 2 h at an emission wavelength (Em) of 520 nm and an (Ex) excitation wavelength of 485 nm, using a BMG lab technologies’ fluorescence plate reader (Offenburg, Germany). The ORAC values were measured by calculating the net area under the decay curves (AUC) and were expressed as µmol TE/g dw.

### 3.7. Cellular Antioxidant Activity

#### 2′,7′-Dichlorodihydrofluorescein Diacetate (DCFH-DA) Assay

DFCHA-DA assay was performed according to Serem [56]. The mouse fibroblast (L929) and human colon adenocarcinoma (Caco-2) cells were plated at 4 × 10^3^ cells/100 µL in a 96 well plate and were cultured for a further 16 h at 37 °C at 5% CO_2_ before the determination of cellular antioxidant activity (CAA). Briefly, 50 µL of 75 µM dichlorofluorescein diacetate (DCFH-DA), final concentration 25 µM solution, was pipetted to each well and incubated at 37 °C for an hour. The medium was then discarded, and the cells were washed once with PBS before being exposed to a 50 µL sample followed by 50 µL of 0.368 mM AAPH. The change in fluorescence was then measured every 2 min for 1 h at an Em of 520 nm and an Ex of 485 (BMG lab technologies, Offenburg, Germany, fluorescence plate reader). The gradient of change in fluorescence was measured, and the % oxidative damage (% OD) was calculated as follows.

% OD = [(Sample − control PBS)/(AAPH − control PBS)] × 100(3)

### 3.8. Macromolecule Protective Ability

#### 3.8.1. Copper-Mediated LDL Oxidation

The copper-mediated LDL oxidation assay was performed according to Antoni et al. [57]. Briefly, sequentially, 100 µL volume of LDL (100 µg/mL protein) was added to 10 µL, 1 mg/mL sample, followed by 10 µL 0.1 M PBS and 10 µL of a 55 µM copper sulphate solution, and then the mixture was incubated for 16 h at 37 °C. Negative control samples contained 100 µL LDL and 30 µL 0.1 M PBS, whereas positive controls contained 100 µL of LDL, 10 µL of copper sulphate, and 20 µL of PBS. After incubation, the following were added sequentially, 10 µL of 1 mM BHT, 60 µL of a Hepes buffered solution (5 mM), 50 µL of a 10% TCA and 75 µL of a 1% TBA solution. The mixture was incubated for 2 h in a 60 °C water bath before centrifuging the samples at 1271× *g* for 3 min. The fluorescence of 75 µL supernatant was measured at an Em of 590 nm and an Ex of 544 nm (BMG lab technologies fluorescence plate reader, Offenburg, Germany). Results were expressed as µmol Malondialdehyde (MDA)/g dw.

#### 3.8.2. Inhibition of Oxidative DNA Damage

The method by Kayitesi [58] was used to determine DNA protection. A mixture containing 5 µL sample, 5 µL of a 15 µg/mL PBR322 plasmid solution and 5 µL 3.69 mM AAPH was incubated at 37 °C for 1 h. Controls without AAPH were also prepared and contained 5 µL, 0.1 M PBS. A 1% agarose gel in 1X Tris-acetate EDTA (TAE) buffer containing 1% ethidium bromide was used to separate the supercoiled and linear forms of the plasmid for 2 h at 60 V. Image processing Gel-Pro Analyzer version 3.0 software supplied by Media Cybernetics, (USA) was used for gel imaging and data analysis.

### 3.9. Statistical Analysis

Statistica software, version 10.0 (Statsoft, Tulsa, OK, USA) was used to analyse data. Three-way analysis of variance (ANOVA) and Tukey’s post hoc test was used to analyse differences between extraction methods, method of processing, and plant species. A significant difference was considered when *p* < 0.05. Pearson’s correlation test was used to analyse the association between TPC, TFC, and antioxidant activity. A *p*-value of 1 until 0.8 was considered a very strong correlation and 0.8 to 0.6 a moderate; only very strong correlations were discussed [59]. The means and standard deviations from all experiments were results of at least two experiments performed in triplicate n = 6. Principal component analysis (PCA) was performed using the XLSTAT statistical and data analysis solution (Boston, MA, USA).

## 4. Conclusions

In this study, the effects of boiling and in vitro digestion of phenolic compounds and bioactivity of African pumpkin and spinach were demonstrated. The majority of the phenolic metabolites profiled were dominant in the undigested extract of the boiled African pumpkin leaves as well as the filtrate. Lower concentrations of profiled phenolics were observed in the digested extracts, indicating the partial extraction and degradation of phenolics during digestion due to changes in pH of the gastrointestinal tract and action of enzymes. However, the bioactivity of the digested extracts was maintained, particularly in the physiologically relevant Caco-2 cells, where AAPH oxidative damage was prevented. Although none of the African pumpkin leaf extracts prevented the oxidative damage of LDL, the protection of DNA was observed. The health benefits of the African pumpkin and spinach demonstrated in this study are due to the reduction of oxidation in cells and the inhibition of the damage of DNA, potentially reducing the risk of cancer development. The bioactivity observed after cooking and in vitro digestion of African pumpkin was comparable to that of spinach, therefore, we suggest that there may be benefits to promoting the increased use of African pumpkin for the maintenance of health.

## Figures and Tables

**Table 1:** Compounds identified in African pumpkin undigested (raw and boiled) extracts and digested (raw and boiled) extracts.

Rt	Precursor Ion [M − H]^−^	Formula [M − H]^−^	Diagnostic Fragments	Uvmax	Compound Name	Effect of Boiling *		Effect of Digestion *	
						RV	BFV	RV	BFV
1.71	191.06	C_7_H_12_O_6_	127, 111, 93, 85	210	Quinic acid	0.13 ± 0.01	3.38 ± 0.15	0.61 ± 0.14	0.46 ± 0.04
9.86	353.09	C_16_H_17_O_9_	191, 179, 173, 135	326	3-Caffeoylquinic acid	16.40 ± 0.61	11.97 ± 0.39	ND	0.83 ± 0.03
11.36	289.07	C_15_H_13_O_6_	245, 205, 203, 123, 109	278	Catechin	79.21 ± 8.62	1.15 ± 0.19	6.07 ± 0.24	1.13 ± 0.44
11.42	353.09	C_16_H_17_O_9_	191, 179,173,135	326	4-Caffeoylquinic acid	ND	ND	ND	0.77 ± 0.07
12.41	353.09	C_16_H_17_O_9_	191, 179, 173, 135	326	5-Caffeoylquinic acid	36.64 ± 0.29	52.56 ± 3.63	ND	ND
16.57	317.24	C_15_H_9_O_8_	315	255, 375	Myricitin	ND	22.38 ± 1.55	2.64 ± 0.68	7.87 ± 0.39
16.87	609.15	C_27_H_29_O_16_	300, 301, 271, 255	257, 353	Quercetin-3-*O*-robinobioside	1.87 ± 0.16	2.50 ± 0.09	0.76 ± 0.03	0.71 ± 0.01
17.14	609.15	C_27_H_29_O_16_	300, 301, 271, 255	353	Rutin	6.84 ± 0.12	2.85 ± 0.30	1.26 ± 0.03	0.68 ± 0.05
17.64	463.09	C_21_H_19_O_12_	300, 301, 271, 255	348	Quercetin-3-*O*-glucoside	111.70 ± 1.66	122.08 ± 0.40	ND	0.81 ± 0.01
18.9	491	C_23_H_23_O_12_	285, 151, 135	307	Unknown-kaempferol 1	ND	3.68 ± 0.57	5.05 ± 0.04	ND
19.01	515.12	C_25_H_23_O_12_	353, 179, 173	325	3,4-Dicaffeoylquinic acid	ND	47.71 ± 6.86	ND	0.77 ± 0.07
19.69	515.12	C_25_H_23_O_12_	353, 179, 173	325	3,5-Dicaffeoylquinic acid	24.68 ± 1.08	62.27 ± 2.85	1.28 ± 0.04	1.61 ± 0.02
20.84	515.12	C_25_H_23_O_12_	353, 179, 173	325	4,5-Dicaffeoylquinic acid	68.93 ± 13.1	110.64 ± 12.2	1.94 ± 0.09	ND
22.35	491	C_23_H_23_O_12_	285, 151, 135	307	Unknown-kaempferol 2	3.72 ± 0.31	7.99 ± 0.25	1.13 ± 0.12	1.17 ± 0.64
23.86	301.03	C_15_H_9_O_7_	151, 179	256	Quercetin	15.94 ± 0.13	2.19 ± 0.21	3.60 ± 0.05	0.77 ± 0.08

Rt—Retention time, ND—Not Detected, RV—Raw vegetable, BFV—Boiled Filtered Vegetable. * Phenolic compound concentration expressed as µg/g dry weight rutin equivalents.

**Table 2:** Total phenolic contents (TPC), total flavonoid content (TFC), and % bioaccessibility (% BA) of African pumpkin and spinach.

Samples	TPC mg GAE/g dw	TFC mg QE/g dw	TPC% BA	TFC% BA
African pumpkin (African pumpkin)				
Undigested				
RV	64.53 ± 9.1 ^bc^	69.00 ± 1.3 ^i^	-	-
BFV	60.41 ± 15 ^bc^	44.34 ± 1.2 ^gh^	-	-
BV-NF	52.27 ± 2.0 ^b^	63.90 ± 5.6 ^i^	-	-
Oral digestion				
RV	37.13 ± 0.9 ^ab^	4.33 ± 0.3 ^a^	57.54	6.27
BFV	43.38 ± 1.9 ^b^	18.44 ± 7.2 ^b^	71.81	41.58
BV-NF	53.83 ± 13 ^b^	25.31 ± 3.7 ^bcd^	102.98	39.61
Gastric digestion				
RV	37.42 ± 4.3 ^ab^	30.22 ± 0.2 ^cde^	57.99	43.94
BFV	40.98 ± 5.5 ^b^	28.43 ± 2.4 ^cd^	67.83	64.12
BV-NF	42.58 ± 1.5 ^b^	64.69 ± 16 ^i^	81.46	101.24
Duodenal digestion				
RV	44.82 ± 2.5 ^b^	50.07 ± 3.7 ^h^	69.46	72.57
BFV	44.55 ± 3.4 ^b^	39.20 ± 6.5 ^efg^	73.74	88.40
BV-NF	4.03 ± 0.7 ^a^	3.50 ± 3.6 ^a^	7.71	5.48
Digestion effect on				
RV	Unchanged	Reduced		
BFV	Unchanged	Unchanged		
BV-NF	Reduced	Reduced		
Spinach				
Undigested				
RV	349.38 ± 7.8 ^h^	133.09 ± 2.6 ^k^	-	-
BFV	279.10 ± 1.7 ^fg^	93.41 ± 0.89 ^j^	-	-
BV-NF	289.78 ± 4.4 ^g^	93.82 ± 2.7 ^j^	-	-
Oral digestion				
RV	248.58 ± 4.8 ^fg^	69.14 ± 3.2 ^i^	71.14	51.94
BFV	115.18 ± 5.2 ^d^	23.76 ± 1.4 ^bcd^	41.27	25.44
BV-NF	128.15 ± 3.9 ^d^	42.11 ± 2.1 ^fgh^	44.22	44.88
Gastric digestion				
RV	260.03 ± 2.4 ^fg^	48.83 ± 4.7 ^gh^	74.43	36.69
BFV	201.68 ± 2.9 ^e^	33.76 ± 0.18 ^def^	72.26	36.14
BV-NF	401.21 ± 2.9 ^h^	33.17 ± 4.2 ^k^	138.45	35.35
Duodenal digestion				
RV	124.25 ± 8.9 ^d^	29.06 ± 3.13 ^cd^	35.56	21.83
BFV	93.88 ± 6.0 ^cd^	16.44 ± 2.1 ^b^	33.64	17.60
BV-NF	69.01 ± 2.7 ^bc^	17.37 ± 1.3 ^b^	23.81	18.51
Digestion effect on				
RV	Reduced	Reduced	-	-
BFV	Reduced	Reduced	-	-
BV-NF	Reduced	Reduced	-	-

Results expressed as dry matter weight (dw), GAE—gallic acid equivalent, QE—quercetin equivalent, TPC—total phenolic contents, TFC—total flavonoid content. All data reported as means ± SD (n = 6) of 2 experiments done in triplicate. Alphabets in superscripts within a column show significant differences at *p* < 0.05. Raw vegetable (RV), boiled filtered vegetable (BFV), boiled vegetable–not filtered (BV-NF).

**Table 3:** Radical scavenging activity/redox potential of African pumpkin and spinach.

Samples	TEAC µM TE/g dw	ORAC µM TE/g dw	FRAP µM TE/g dw
African pumpkin			
Undigested extract			
RV	216.50 ± 37 ^bcd^	531.29 ± 5.7 ^cd^	50.14 ± 6.0 ^b^
BFV	211.50 ± 21 ^bcd^	406.66 ± 12 ^bc^	48.36 ± 11 ^b^
BV-NF	262.11 ± 17 ^bcde^	340.46 ± 4.0 ^bc^	148.78 ± 16 ^ef^
Oral digestion			
RV	221.08 ± 6.2 ^bcd^	450.56 ± 1.1 ^bc^	88.62 ± 1.0 ^cd^
BFV	290.85 ± 1.8 ^cde^	429.60 ± 10 ^bc^	45.68 ± 5.6 ^b^
BV-NF	298.35 ± 7.1 ^de^	193.87 ± 0.3 ^a^	60.18 ± 0.7 ^bc^
Gastric digestion			
RV	154.84 ± 37 ^b^	497.99 ± 6.4 ^bc^	52.34 ± 15 ^b^
BFV	167.76 ± 23 ^b^	483.05 ± 7.6 ^bc^	47.83 ± 3.9 ^b^
BV-NF	184.42 ± 17 ^bc^	186.57 ± 1.3 ^a^	40.91 ± 2.5 ^b^
Duodenal digestion			
RV	179.00 ± 23 ^b^	283.53 ± 6.0 ^ab^	47.43 ± 7.8 ^b^
BFV	193.79 ± 9 ^bcd^	326.53 ± 2.1 ^bc^	116.93 ± 1.2 ^de^
BV-NF	−203.21 ± 20 ^a^	191.30 ± 0.1 ^a^	−36.92 ± 4.8 ^a^
Digestion effect on			
RV	Unchanged	Reduced	Unchanged
BFV	Unchanged	Unchanged	Increased
BV-NF	Reduced	Reduced	Reduced
Spinach			
Undigested extract			
RV	1090.99 ± 1.4 ^i^	756.20 ± 1.5 ^d^	334.16 ± 4.5 ^k^
BFV	1021.96 ± 2.4 ^i^	1223.21 ± 9.7 ^ef^	263.46 ± 0.96 ^j^
BV-NF	1084.84 ± 1.5 ^i^	1181.94 ± 2.5 ^ef^	351.83 ± 2.9 ^k^
Oral digestion			
RV	712.53 ± 9.8 ^g^	637.67 ± 3.7 ^f^	172.38 ± 2.8 ^fg^
BFV	495.93 ± 9.3 ^f^	1363.33 ± 1.7 ^f^	190.73 ± 1.7 ^gh^
BV-NF	370.37 ± 3.3 ^e^	540.65 ± 4.3 ^cd^	184.62 ± 0.96 ^g^
Gastric digestion			
RV	857.14 ± 7.0 ^h^	1681.07 ± 5.2 ^g^	228.12 ± 6.7 ^i^
BFV	748.83 ± 5.8 ^gh^	1179.40 ± 7.2 ^ef^	139.07 ± 5.8 ^e^
BV-NF	1090.99 ± 9.3 ^i^	546.13 ± 4.6 ^cd^	249.86 ± 2.7 ^ij^
Duodenal digestion			
RV	534.01 ± 6.6 ^f^	1000.71 ± 1.2 ^e^	226.76 ± 2.9 ^i^
BFV	523.30 ± 5.9 ^f^	1004.29 ± 7.8 ^e^	218.60 ± 2.9 ^hi^
BV-NF	172.21 ± 1.5 ^b^	563.79 ± 2.2 ^cd^	122.76 ± 1.5 ^e^
Digestion effect on			
RV	Reduced	Increased	Reduced
BFV	Reduced	Unchanged	Unchanged
BV-NF	Reduced	Reduced	Reduced

Results expressed as dry matter weight (dw), µM TE—micromolar Trolox equivalent, TEAC—Trolox equivalence antioxidant capacity, ORAC—oxygen radical antioxidant capacity, FRAP—ferric reducing antioxidant power. All data reported as means ± SD (n = 6) of 2 experiments done in triplicate. Alphabets in superscripts within a column show significant differences at *p* < 0.05. Raw vegetable (RV), boiled filtered vegetable (BFV), boiled vegetable—not filtered (BV-NF).

**Table 4:** Cellular antioxidant activity of African pumpkin and spinach.

Samples	% Oxidative Damage DCFH-DA_L929_	% Oxidative Damage DCFH-DA_Caco-2_
African pumpkin		
Undigested extract	Control (AAPH + L929 cells + PBS) 100%	Control (AAPH + Caco-2 cells + PBS) 100%
RV	−16.54 ± 12 ^a^	−8.88 ± 8.9 ^ab^
BFV	−11.84 ± 3.8 ^ab^	−6.50 ± 1.1 ^ab^
BV-NF	−15.50 ± 6.1 ^a^	−10.91 ± 1.3 ^a^
Oral digestion		
RV	78.39 ± 5.6 ^d^	515.25 ± 6.0 ^h^
BFV	8.77 ± 1.5 ^b^	29.96 ± 4.5 ^f^
BV-NF	−5.17 ± 5.1 ^ab^	−0.96 ± 1.3 ^abcd^
Gastric digestion		
RV	55.65 ± 3.6 ^c^	−13.83 ± 2.0 ^a^
BFV	−13.45 ± 5.5 ^ab^	6.40 ± 0.1 ^bcde^
BV-NF	−9.22 ± 0.1 ^ab^	−5.65 ± 0.6 ^ab^
Duodenal digestion		
RV	64.22 ± 0.7 ^cd^	−0.90 ± 1.7 ^abcd^
BFV	114.93 ± 0.8 ^e^	68.76 ± 2.3 ^g^
BV-NF	−2.93 ± 6.2 ^ab^	−4.46 ± 1.6 ^abcd^
Digestion effect on		
RV	Increased	Unchanged
BFV	Increased	Increased
BV-NF	Unchanged	Unchanged
Spinach		
Undigested extract		
RV	−21.52 ± 4.7 ^a^	−5.39 ± 2.7 ^abc^
BFV	−20.33 ± 4.1 ^a^	−13.39 ± 6.8 ^a^
BV-NF	−20.57 ± 4.8 ^a^	−10.71 ± 1.0 ^a^
Oral digestion		
RV	102.05 ± 1.0 ^e^	16.49 ± 0.8 ^ef^
BFV	−6.86 ± 3.7 ^ab^	10.19 ± 1.1 ^cde^
BV-NF	−7.22 ± 4.1 ^ab^	6.64 ± 0.3 ^bcde^
Gastric digestion		
RV	−0.006 ± 7.5 ^ab^	−0.83 ± 0.2 ^abcd^
BFV	−20.59 ± 5.1 ^a^	−0.05 ± 0.04 ^abcd^
BV-NF	−10.21 ± 6.1 ^ab^	10.74 ± 1.9 ^de^
Duodenal digestion		
RV	−3.19 ± 0.02 ^ab^	−10.90 ± 5.1 ^a^
BFV	−3.09 ± 0.8 ^ab^	−5.26 ± 2.7 ^bcde^
BV-NF	−5.61 ± 1.4 ^ab^	−7.16 ± 1.1 ^ab^
Digestion effect on		
RV	Unchanged	Unchanged
BFV	Unchanged	Increased
BV-NF	Unchanged	Unchanged

OD-oxidative damage, DCFH-DA—2′,7′-dichlorodihydrofluorescein diacetate, L929—mouse fibroblast, Caco-2—human epithelial colorectal adenocarcinoma. Raw vegetable (RV), boiled filtered vegetable (BFV), boiled vegetable—not filtered (BV-NF), Phosphate-buffered solution (PBS). Alphabets in superscripts within a column show significant differences at *p* < 0.05.

## Data Availability

The data presented in this study are contained within the article and are also available in the Supplementary Materials.

## Supplementary Materials

The following are available online, Table S1: Pearson correlation coefficient (r) between investigated parameters in African pumpkin and spinach samples and Figure S1: Sample preparation procedure to determine the effects of boiling.

## Sample Availability

Samples of the compounds are not available from the authors.
