# Supplementary material for: Hydrothermal Processing and In Vitro Simulated Human Digestion Affects the Bioaccessibility and Bioactivity of Phenolic Compounds in African Pumpkin (Momordica balsamina) Leaves
Source: Molecules. 2021 Aug 27;26(17):5201. doi: 10.3390/molecules26175201 (PMC8434164; doi:10.3390/molecules26175201)
Supplement: Supplementary file 1 [file molecules-26-05201-s001.zip › molecules-1338022-supplementary.pdf]

Sample preparation

Vegetables used in this study were sampled from Tzaneen, Limpopo region, SA. Professor A. Moteetee, a herbarium curator from the Department of Botany, University of Johannesburg, confirmed the vegetable species. After harvesting, the vegetables were washed and chopped. Samples generated were prepared as shown in Supplementary Fig S1 and S2. Freeze-drying was done at -55° C for 24 h.

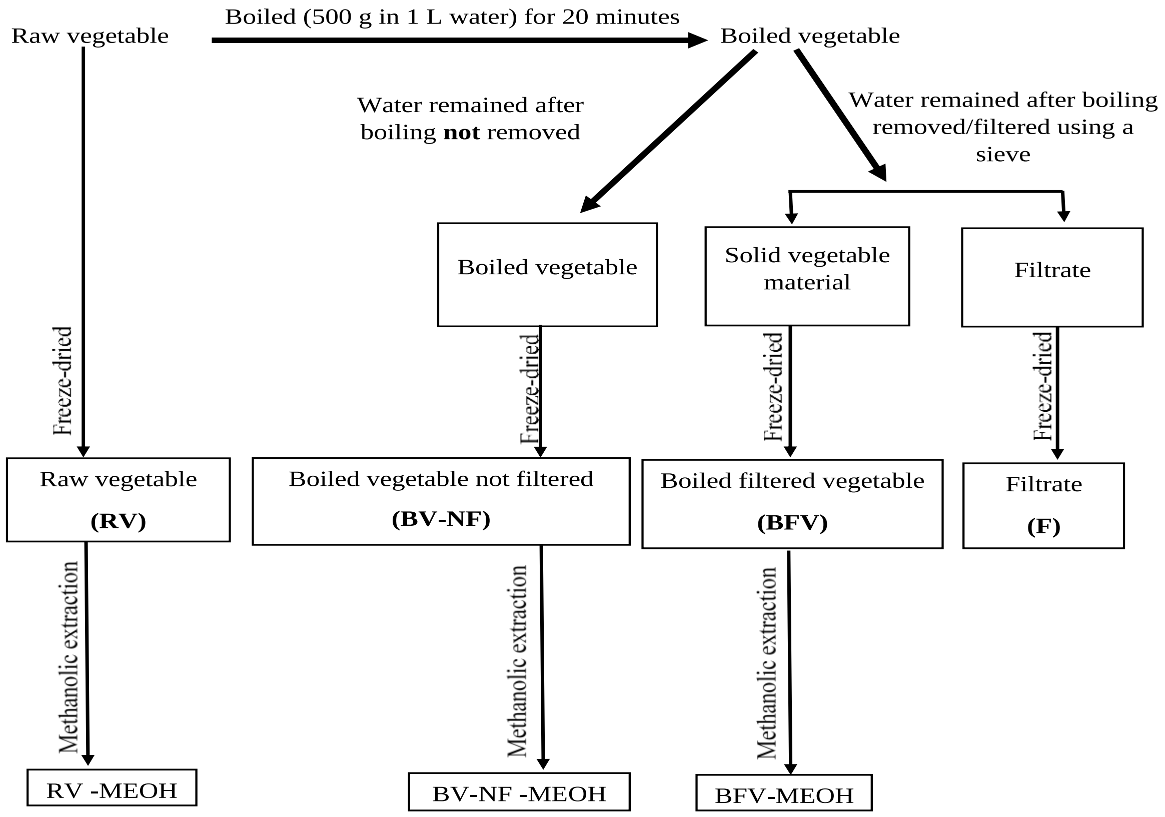

Supplementary Figure S1: Sample preparation procedure to determine the effects of boiling

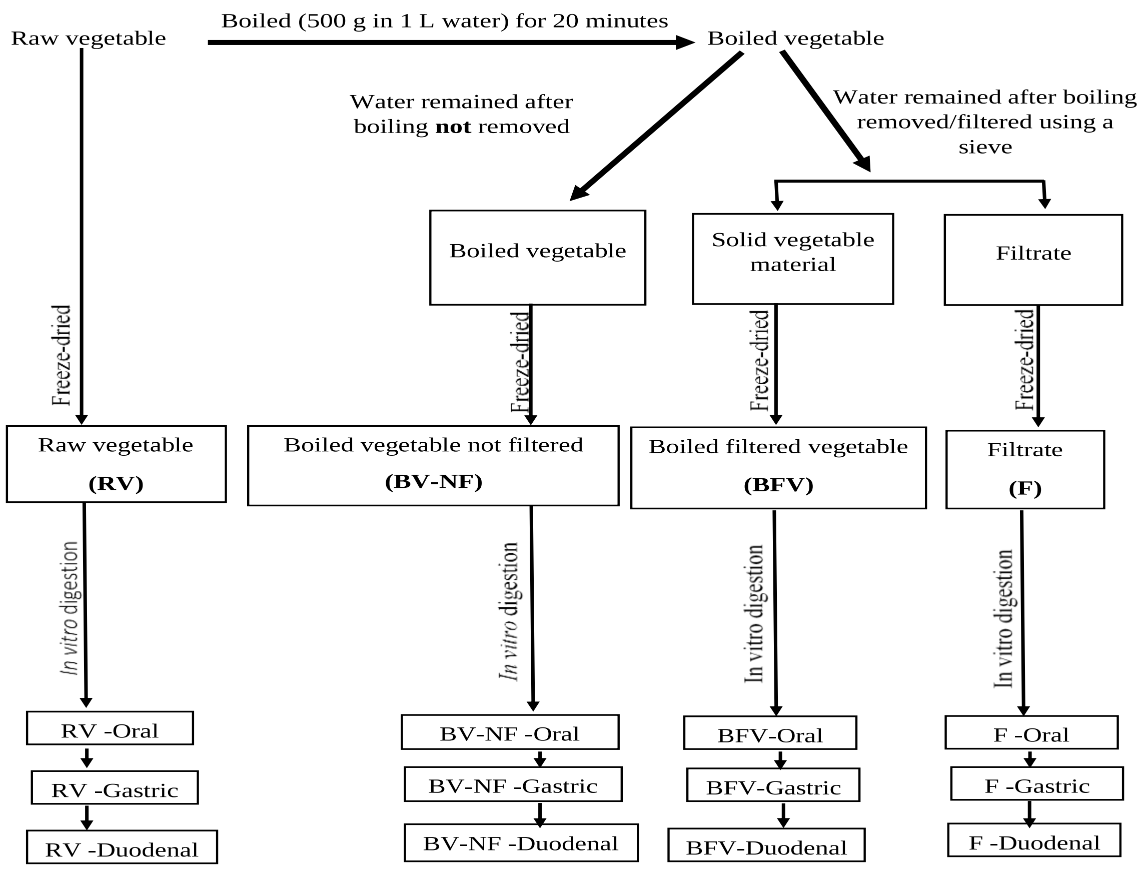

Supplementary Figure S2: Sample preparation procedure to determine the effects of *in vitro* digestion.

Supplementary Table S1: Pearson correlation coefficient (r) between investigated parameters in African pumpkin and spinach samples

	**African pumpkin**							**Spinach**						
	**TPC**	**TFC**	**TEAC**	**ORAC**	**FRAP**	**%OD DCFH-DA$_{L929}$**	**%OD DCFH-DA$_{Caco-2}$**	**TPC**	**TFC**	**TEAC**	**ORAC**	**FRAP**	**%OD DCFH-DA$_{L929}$**	**%OD DCFH-DA$_{Caco-2}$**
**Methanolic**														
TPC	1.000	**0.943**	*0.771*	*-0.657*	0.314	-0.086	0.543	1.000	0.486	*0.714*	*-0.771*	0.371	*-0.714*	0.429
TFC		1.000	**0.829**	*-0.771*	0.257	-0.029	0.429		1.000	0.371	*-0.600*	0.543	-0.029	0.257
TEAC			1.000	*-0.771*	*0.600*	0.371	-0.086			1.000	*-0.771*	*0.771*	*-0.714*	0.371
ORAC				1.000	0.029	-0.314	-0.200				1.000	*-0.600*	0.314	-0.429
FRAP					1.000	0.200	-0.486					1.000	-0.257	-0.143
**Oral digestion**														
TPC	1.000	0.314	-0.543	0.143	0.314	0.029	0.543	1.000	1.000	0.371	-0.086	-0.200	0.543	0.486
TFC	0.314	1.000	0.029	0.029	**1.000**	*0.600*	0.543		1.000	0.371	-0.086	-0.200	0.543	0.486
TEAC			1.000	-0.371	0.029	-0.429	*-0.657*			1.000	*0.771*	-0.143	**0.829**	**0.886**
ORAC				1.000	0.029	*0.600*	*0.714*				1.000	0.257	*0.714*	0.543
FRAP					1.000	*0.600*	0.543					1.000	-0.200	-0.143
**Gastric digestion**														
TPC	1.000	0.232	-0.314	*-0.771*	0.086	*-0.600*	*0.657*	1.000	**1.000**	**0.886**	-0.429	0.657	0.486	0.486
TFC		1.000	0.348	*-0.638*	*-0.638*	0.319	-0.377		1.000	**0.886**	-0.429	0.657	0.486	0.486
TEAC			1.000	-0.143	-0.486	-0.143	-0.029			1.000	-0.543	0.829	0.486	0.486
ORAC				1.000	0.143	0.371	-0.314				1.000	-0.257	0.429	**-0.943**
FRAP					1.000	-0.314	0.200					1.000	0.486	0.200
**Duodenal**														
TPC	1.000	*0.771*	0.486	0.486	*0.657*	*0.714*	*0.600*	1.000	*0.714*	*0.714*	*0.771*	*0.771*	*0.771*	-0.200
TFC		1.000	0.429	0.429	0.429	0.486	0.371		1.000	0.257	0.314	0.371	0.314	-0.543
TEAC			1.000	1.000	**0.829**	*0.771*	0.429				0.429	*0.600*	0.429	0.086
ORAC				1.000	**0.829**	*0.771*	0.429				1.000	**0.886**	**1.000**	0.143
FRAP					1.000	**0.829**	*0.771*					1.000	**0.886**	0.371

*Bold figures represent very strong significant correlations and figures in italics represent moderate correlations. OD - oxidative damage, DCFH-DA - 2′,7′-dichlorodihydrofluorescein diacetate, L929 - mouse fibroblast, Caco-2 - human epithelial colorectal adenocarcinoma, TEAC - Trolox equivalence antioxidant capacity, ORAC - oxygen radical antioxidant Capacity, FRAP - ferric reducing antioxidant power.
